# Comparing the MRI Appearance of the Lymph Nodes and Spleen in Wild-Type and Immuno-Deficient Mouse Strains

**DOI:** 10.1371/journal.pone.0027508

**Published:** 2011-11-09

**Authors:** Vasiliki Economopoulos, Jennifer C. Noad, Shruti Krishnamoorthy, Brian K. Rutt, Paula J. Foster

**Affiliations:** 1 Department of Medical Biophysics, University of Western Ontario, London, Ontario, Canada; 2 Imaging Research Group, Robarts Research Institute at the University of Western Ontario, London, Ontario, Canada; 3 Radiology Department, Richard M. Lucas Center for Imaging, Stanford University, Stanford, California, United States of America; University of California, San Francisco, United States of America

## Abstract

The goal of this study was to investigate the normal MRI appearance of lymphoid organs in immuno-competent and immuno-deficient mice commonly used in research. Four mice from each of four different mouse strains (nude, NOG, C57BL/6, CB-17 SCID (SCID)) were imaged weekly for one month. Images were acquired with a 3D balanced steady state free precession (bSSFP) sequence. The volume of the lymph nodes and spleens were measured from MR images. In images of nude and SCID mice, lymph nodes sometimes contained a hyperintense region visible on MRI images. Volumes of the nodes were highly variable in nude mice. Nodes in SCID mice were smaller than in nude or C57Bl/6 mice (p<0.0001). Lymph node volumes changed slightly over time in all strains. The spleens of C57Bl/6 and nude mice were similar in size and appearance. Spleens of SCID and NOG mice were significantly smaller (p<0.0001) and abnormal in appearance. The MRI appearance of the normal lymph nodes and spleen varies considerably in the various mouse strains examined in this study. This is important to recognize in order to avoid the misinterpretation of MRI findings as abnormal when these strains are used in MRI imaging studies.

## Introduction

Immuno-deficient mice are routinely used in research. These mice have a limited capacity for rejecting foreign tissue, which makes them excellent recipients for xenografts of human cells and tissues [1]. A variety of genetic mutations are known that impair immune function in mice. Genetic loci affecting immune responses include nu (nude), SCID (severe combined immunodeficiency), beige, and xid (X-linked immunodeficiency) [2]. The various mouse mutants have differing immunological properties. The extent to which some of these mutations interfere with immune function can also vary with the genetic background.

Some commonly used strains of immuno-deficient mice include the nude mouse, the severe combined immune deficiency (SCID) mouse and the NOD/SCID mouse (non-obese diabetic/SCID). The murine recessive nude mutation on chromosome 11 arose spontaneously [2]. Homozygotes (nu/nu) are hairless from birth and completely lack a thymus due to a failure of development of the thymic cells at the embryonic stage [2], [3]. The lack of the thymus leads to many defects of the immune system, including a greatly reduced population of T lymphocytes.

The murine recessive SCID mutation on chromosome 16 arose in the CB-17 inbred strain (BALB/c.C57BL/Ka-Igh-1b). Homozygotes lack both B and T cells but have normal numbers of natural killer (NK) cells, the main effectors of non-MHC restricted immunity [2]. In 1995, Shultz et al. described a new immuno-deficient mouse model, the NOD/SCID, obtained by crossing the SCID and NOD mouse strains [4]. The NOD strain is characterized by functional deficits in NK cells, an absence of circulating complement and defects in the differentiation and function of antigen-presenting cells [5]. The NOD/SCID model combines multiple functional defects of adaptive and innate immunity. They are very suitable for xenografts of human cell lines.

The NOD/SCID IL2Rγ null (NOG) mouse is a relatively new immuno-deficient mouse established in an attempt to generate a more appropriate recipient for xenotransplantation. NOG mice are NOD/SCID mice that have an additional mutation in the common gamma chain of the IL2 receptor (IL2Rγ) [6]. In addition to lacking functional T and B lymphocytes, the IL2Rγ deficiency blocks the development of NK cells. The NOG mice have no ‘leakiness’ with age, meaning that NK cells are not ever produced. The NOG mouse accepts heterologous cells much more easily compared with any other type of immuno-deficient mouse. Thus, the NOG mouse is currently viewed as the most sensitive mouse model to allow human cells to engraft, proliferate, and/or differentiate [7].

The nude mouse has been widely studied in cancer research [8], [9]. Xenografts of many different established human cancer cell lines have been successfully grown in nude mice after their implantation in subcutaneous or orthotopic sites [10], [11]. However, the engraftment and growth rates, and the incidence of metastases, are often enhanced in the other more immuno-deficient mouse strains [1], [2], [12]–[14]. A comparative study of tumor growth in various cancer cell lines in different mouse strains (nude, C.B.-17 SCID (SCID) and NOD/SCID mice) has shown a better growth rate in the NOD/SCID mouse [1]. Taghian et al. compared the transplantability of six human cancer cell lines in nude and SCID mice. For 6/6 cell lines, the number of cells required to establish a tumor was significantly lower for SCID mice compared to nude mice [15]. Xie et al. showed that nude and SCID mice were equally suitable for growing three different human cancer cell lines (bladder, breast and melanoma), but that the metastatic capacity of the cells was much better expressed in the SCID mice [14].

Magnetic resonance imaging (MRI) is becoming increasingly common as a tool for the noninvasive monitoring of disease in a wide range of murine models. Therefore, it is important to understand the baseline MRI appearance of the lymph nodes and spleen in experimental mice. Three strains of immune-compromised mice were chosen (nude, SCID and NOG) because of their frequent use as experimental models in research. Because of the altered structure and function in the lymphoid organs (spleen, lymph nodes) in immuno-deficient mice we hypothesized that there would be noteworthy differences in the normal MRI appearance of the lymph nodes and spleen in immuno-deficient mice compared to immuno-competent mice. In the current study we use MRI to characterize the lymph nodes and spleen in three different immuno-deficient mice (nude, SCID and NOG) and the immune-competent mouse strain C57Bl/6.

## Materials and Methods

All animal experiments were approved by the Animal Use Subcommittee of the University Council on Animal Care at The University of Western Ontario following the guidelines of the Canadian Council on Animal Care (protocol # 2010–210).

### Mice

Mice used included the wild-type C57Bl/6J mice (Jackson Laboratories, age 7 to 9 weeks at arrival) and immuno-deficient nude (nu/nu) mice (Charles River Canada, age 6 to 8 weeks at arrival), CB-17 SCID (CB17/Icr-Prkdc^scid^/IcrIcoCrl) mice (Charles River Canada, age 6 to 8 weeks at arrival) and NOG mice (Jackson Laboratories, age 8 to 11 weeks at arrival). In the first experiment 4 mice per strain were imaged once, within a week of arrival, to compare the MRI appearance of the lymphoid organs. In the second experiment 4 mice per strain (excluding the NOG mice, which the first experiment revealed had no MR visible nodes) were imaged on days 7, 14 and 28 after arrival to assess changes in the appearance of the nodes and spleens over time. The immuno-deficient mice used in this longitudinal imaging experiment left the barrier proper for the first scanning session and returned to an external barrier where they were housed in safe conditions within ventilated cages for the reminder of the study. All animals were sacrificed after the final imaging time point and the tissues of interest were weighed and prepared for histology.

### Magnetic Resonance Imaging

All imaging was performed on a 1.5T CV/I MRI scanner (General Electric Medical Systems, Milwaukee, WI) using a custom built gradient coil insert (inner diameter  = 17.5 cm, maximum gradient strength  = 500 mT/m, and peak slew rate  = 3000 T/m/sec) and a custom built solenoid mouse body radio-frequency (RF) coil (4 cm in length and 3 cm in diameter). All mice were imaged with a 3D balanced steady state free precession (bSSFP) pulse sequence. The bSSFP sequence was chosen because it provides very high SNR efficiency, allowing for high resolution image acquisitions of the whole mouse body in reasonable scan times, and because it produces very good soft tissue contrast, related to T2/T1. The scan parameters for bSSFP were as follows: repetition time = 6.7 ms, echo time = 3.3 ms, flip angle = 40°, bandwidth = +/−31.25 kHz, matrix  = 300×300, field of view (FOV)  = 6 cm, 0.2 mm slice thickness, 200 µm isotropic spatial resolution, 4 signal averages (NEX), 4 RF phase cycles and scan time of 34 minutes.

Since both fat and fluid appear with high signal intensity in bSSFP images spin echo (SE) images were acquired with T1- and T2-weighting in some nude mice. These additional image contrasts helped with the interpretation of the hyperintense regions within some lymph nodes on bSSFP images. The SE parameters were as follows: repetition time  = 2000 ms for T2w and 600 ms for T1w, echo time  = 80 ms for T2 w and 25 ms for T1w, matrix  = 256×256, FOV  = 6 cm FOV, in-plane spatial resolution  = 234 microns, slice thickness = 500 microns, 12 NEX, scan time = ∼15 minutes for T1w and ∼51 minutes for T2w images.

### Image Analysis

The volume of the spleen and the left and right axillary, brachial, inguinal and popliteal lymph nodes were measured by manual segmentation from all of the acquired images. The axillary, brachial, inguinal and popliteal lymph nodes were chosen for comparison since they are easily visualized and are commonly investigated in metastasis and immunotherapy experiments. The Osirix image analysis software [16] was used to make all measurements from images acquired in this study. Each organ of interest was segmented individually on every image slice to create a series of regions of interest (ROI). One ROI from the organ's ROI series was selected and the volume was calculated by using the ROI volume calculation tool available within the software package. This procedure was repeated to calculate the volume for all organs in each image. All volume data were compared statistically using Graph Pad Prism analysis software (GraphPad Software, La Jolla, CA).

### Histopathological Analysis

All animals were euthanized using a carbon dioxide gas chamber. Lymph nodes were then removed and placed in 3.75% formalin. The fixed lymph nodes were imbedded into paraffin blocks and sectioned. Four 5 µm sections were cut from each block, followed by a 1 µm gap and an additional four 5 µm sections. The sections were then stained with Hematoxylin and Eosin (H&E).

## Results

### Lymph Node Appearance and Volumes

**Figure 1:**
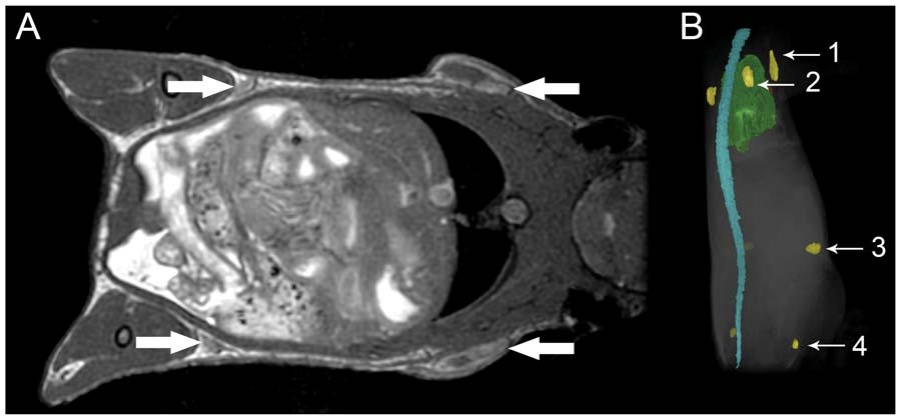
Location of lymph nodes within the mouse body. (A) Whole mouse body bSSFP image of a C57Bl/6 mouse showing both the brachial and inguinal lymph nodes (arrows) and (B) 3D reconstruction showing the location of various lymph nodes within the mouse; 1 – axillary node, 2 – brachial node, 3 – inguinal node, 4 – popliteal node.

In the first set of studies, C57Bl/6, SCID, nude and NOG mice were imaged once. Figure 1 shows coronal views of the whole mouse body and the locations of the axillary, brachial, inguinal and popliteal lymph nodes in bSSFP images of a C57Bl/6 mouse. The images of lymph nodes in the C57Bl/6 mice appear with uniform signal intensity and have good contrast with the surrounding tissue for the brachial, inguinal and popliteal lymph nodes, which are located in fat pads.

**Figure 2:**
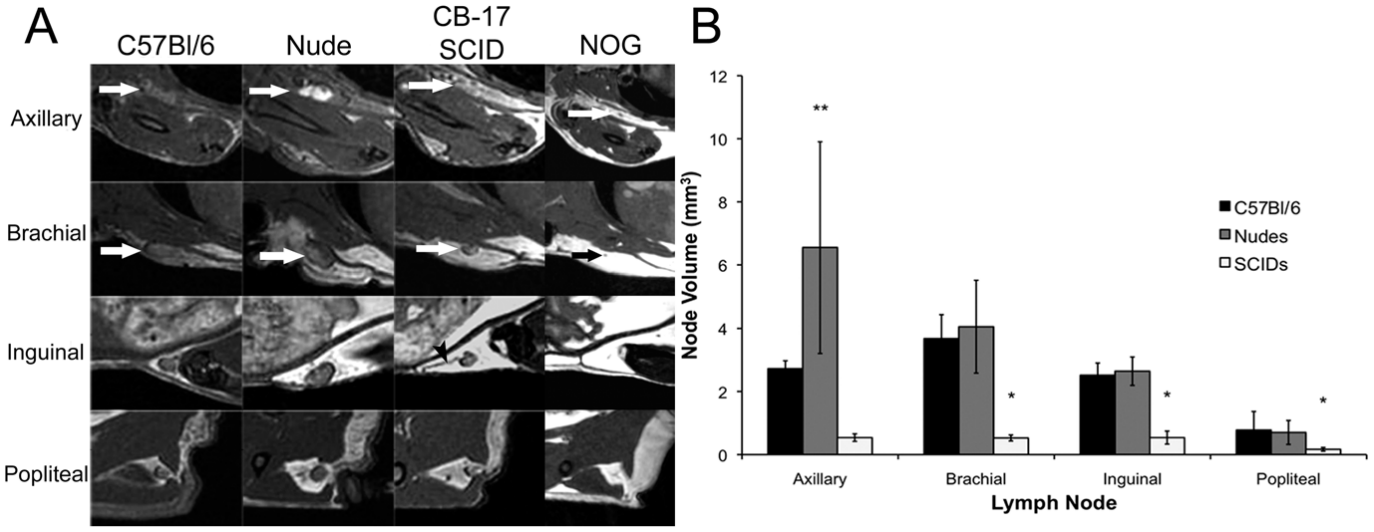
MR appearance and volumes of lymph nodes various mouse strains. (A) MR appearance of the axillary, brachial, inguinal and popliteal lymph nodes in C57Bl/6, Nude, CB-17 SCID and NOG mice. The brachial, inguinal and popliteal lymph nodes are easiest to visualize due to their location within a fat pad. Lymphatic vessels are also visible in acquired images (arrowhead). Images for the NOG mice are included for completeness although there were no MRI detectable lymph nodes. (B) Volumes of the axillary, brachial, inguinal and popliteal lymph nodes in C57Bl/6, Nude and CB-17 SCID mice. The brachial, inguinal and popliteal lymph nodes in CB-17 SCID mice were found to be significantly smaller than those in both C57Bl/6 and Nude mice (*, p<0.0001 for brachial and inguinal and p = 0.0128 for popliteal). The axillary node in Nude mice was significantly larger than those in both CB-17 SCID and C57Bl/6 mice (**, p<0.0001). One way ANOVA test was used. Error bars represent the standard deviation.

Representative images of these nodes in the different mouse strains are shown in Figure 2A. There were some notable differences in the MRI appearance of the lymph nodes in the immune compromised mice. First, in images of some of the lymph nodes in the nude mice a large hyperintense region was visible within the node. This was observed in the axillary (5/8 nodes), brachial (8/8 nodes) and inguinal nodes (6/8 nodes). While the brachial nodes most frequently exhibited these hyperintense areas, these areas were anatomically the most obvious in the axillary nodes. This pattern of hyperintensity was also present in some nodes in SCID mice, but at a lower frequency; in the brachial (3/8 nodes), inguinal (1/8 nodes) and popliteal (2/8 nodes) nodes. Bright spots were never observed in C57Bl/6 mice. No lymph nodes were visible in the images of the NOG mice.

Measuring the lymph node volumes from the 3D MR images also revealed differences between mouse strains (Figure 2B). The mean lymph node volumes measured from images acquired in the first experiment are listed in Table 1. All volumes are listed as the mean +/− standard deviation of the mean. The mean values were compared using a one way Analysis of Variance (ANOVA) test with a Tukey multiple comparison posttest. The brachial, inguinal and popliteal nodes in SCID mice were significantly smaller than those in both nude mice and C57Bl/6 mice (p<0.0001 for brachial and inguinal and p = 0.0128 for popliteal). The axillary nodes in nude mice were significantly larger than those in C57Bl/6 mice (p<0.0001), while the brachial, inguinal and popliteal node volumes were comparable between these two strains. The size of all lymph nodes was most variable in the nude mouse.

**Table 1:** Lymph node volumes of C57Bl/6, Nude and CB-17 SCID mice. All volumes listed as mean ± standard deviation.

Lymph Node	C57Bl/6J	Nude	CB-17 SCID
Axillary Node Volume (mm^3^)	2.73±0.25	6.55±3.35	0.54±0.12
Brachial Node Volume (mm^3^)	3.68±0.76	4.06±1.47	0.53±0.09
Inguinal Node Volume (mm^3^)	2.52±0.39	2.65±0.45	0.54±0.21
Popliteal Node Volume (mm^3^)	0.78±0.59	0.70±0.38	0.17±0.06

**Figure 3:**
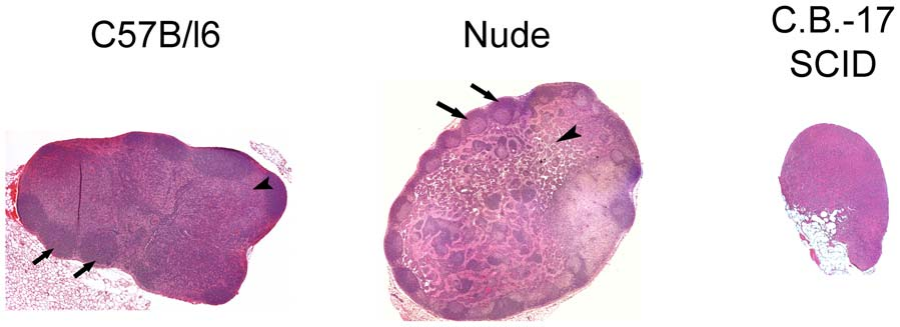
H&E sections of brachial lymph nodes from C57B/l6, Nude and C.B.-17 SCID mice. Whole nodes are shown at 5x magnification. The T cell rich paracortex (arrowsheads) and B cell rich follicles (arrows) can be easily seen in the nodes of C57B/l6 mice, where as in Nude mouse lymph nodes, only the B cell rich follicles can be seen (arrows). In the areas of the paracortex where T cells should be found, vacant areas are detected (arrowheads), helping to explain the hyperintense appearance of many of these nodes in MR images. Nodes in SCID mice lack both the paracortex and follicles, leaving these nodes underdeveloped and significantly smaller in size.

Examination of the H&E staining revealed clear differences in the morphology of nodes from the different mouse strains (Figure 3). In the immune competent C57Bl/6 mice, the nodes contain all the major structures including the cortex, paracortex and medulla. Areas rich in T and B cells are present and fully formed in this strain. Lymph nodes from nude mice have a similar structure; the B cell follicles can easily be distinguished from other structures in the node. However, T cells are lacking in these animals, leaving vacant areas within the paracortex. SCID mouse lymph nodes do not contain any defined structures. The cortex, paracortex and medulla cannot easily be distinguished.

**Figure 4:**
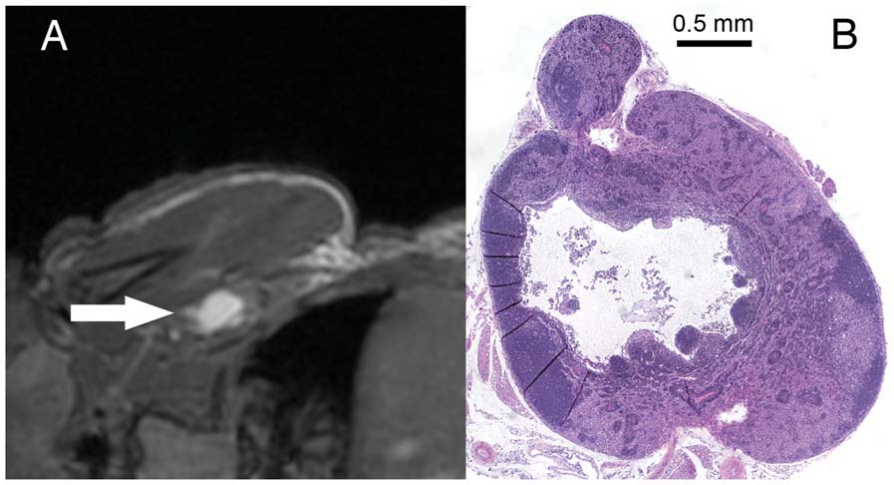
Nude mouse axillary node with hyperintense center. (A) MR image. (B) Corresponding histology. The hyperintense area within the lymph node (arrow) corresponds to a cavity that is visible in the histology.

**Figure 5:**
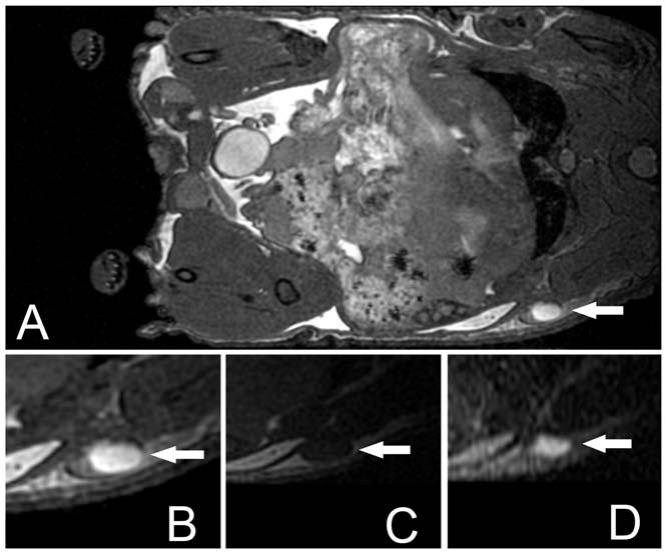
MR images of a nude mouse brachial lymph node acquired with different pulse sequences. (A) bSSFP image of whole mouse body, (B) bSSFP image of lymph node, (C) T1w SE of lymph node (TR = 600 ms, TE = 25 ms), (D) T2w SE of lymph node (TR = 2000 ms, TE = 80 ms)

In some nodes the cavities in the lymph nodes were pronounced. In Figure 4 an H&E stained section of a representative axillary node from a nude mouse is shown along with the corresponding bSSFP image that shows a large region of bright signal within the node. In the T1w images, the central portion of the lymph node had low signal intensity while in the corresponding T2w image the central portion of the node was hyperintense, as in the bSSFP image (Figure 5). Lymph node tissue in NOG mice could not be found upon dissection.

### Spleen Appearance and Volumes

**Figure 6:**
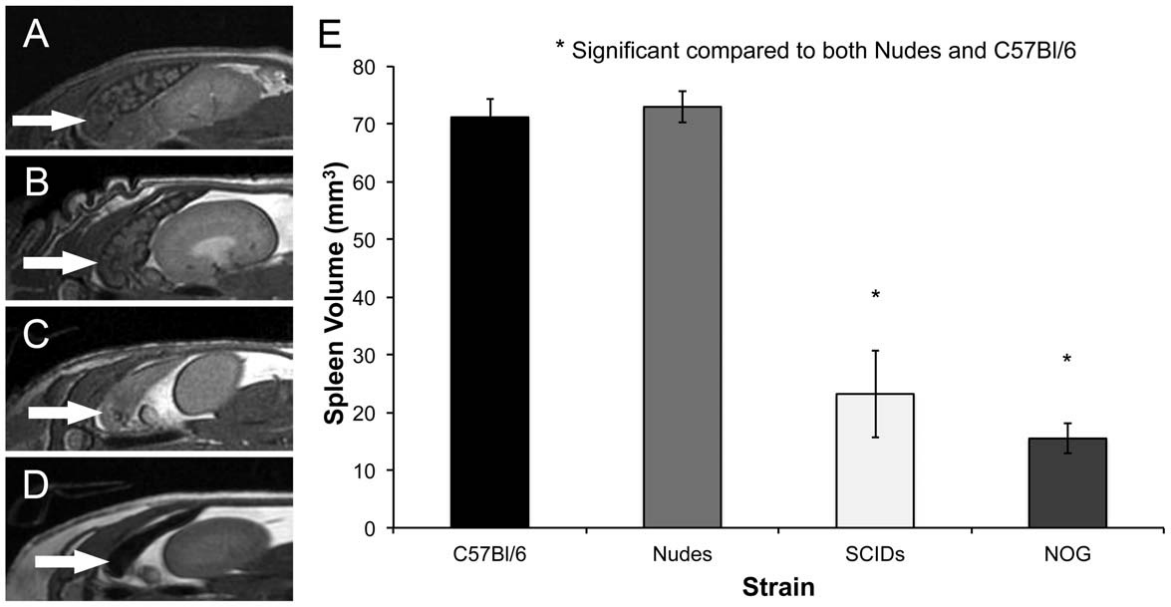
bSSFP images of the spleen. (A) C57Bl/6 mice. (B) Nude mice. (C) CB-17 SCID mice. (D) NOG mice. (E) Spleen volume in C57Bl/6, Nude, CB-17 SCID and NOG mice. (*) Spleen volumes were significantly smaller in CB-17 SCID and NOG mice (p<0.0001) compared to C57Bl/6 and nude mice. One way ANOVA test was used. Error bars represent the standard deviation.

Differences in the MR appearance of the spleen were also observed. The images of the spleen in the C57Bl/6 and nude mice were similar in appearance with a heterogeneous pattern of signal intensities giving it a mottled appearance (Figure 6). The images of the spleen in SCID and NOG mice were visually different. The spleens in SCID mice had a higher signal intensity, which was uniform throughout (6C). The spleens in NOG mice appeared with very low signal intensity, appearing black (6D). The spleen volumes were compared using a one way ANOVA with a Tukey multiple comparison posttest and the average volume of the spleen was found to be significantly smaller (p<0.0001) in both SCID (23.2+/−3.6 mm^3^) and NOG (15.5+/−1.3 mm^3^) mice compared to the both nude (72.9+/−1.4 mm^3^) and C57Bl/6 (71.1+/−1.6 mm^3^) strains (7E).

### Changes in Lymph Node Volumes Over Time

**Figure 7:**
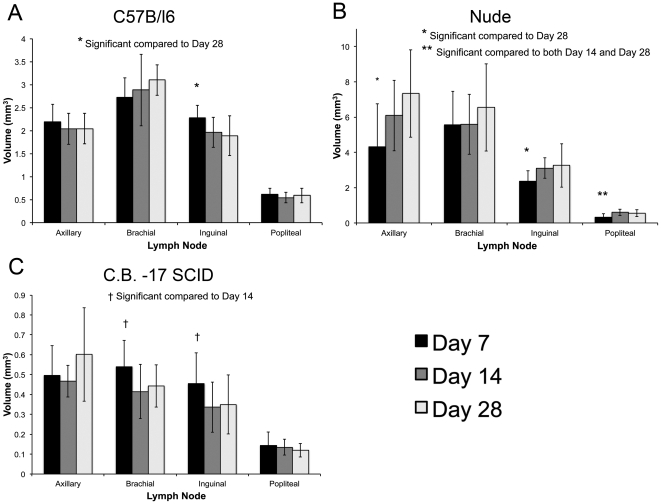
Lymph node volumes over time. (A) C57Bl/6 mice. (B) Nude mice. (C) CB-17 SCID mice. Significant differences were found in the inguinal (p = 0.0071) nodes of C57Bl/6 mice. Significant differences were also found in the axillary (p = 0.0104), inguinal (p = 0.0155) and popliteal (p = 0.0046) nodes of nude mice. Significant differences were also found in SCID mice in the brachial (p = 0.0130) and inguinal nodes (p = 0.0225). (*) Significantly different compared to day 7. Repeated measures ANOVA test was used. Error bars represent the standard deviation.

We next wanted to determine whether there were changes in the MR appearance of lymph nodes over time. C57Bl/6, nude and SCID mice were imaged on days 7, 14 and 28 after arrival. The lymph node volumes were measured from images acquired at each time point (Figure 7). The lymph node volumes were compared over time using a repeated measures ANOVA test with a Tukey multiple comparison posttest. In all mice there were changes in the lymph node volumes over time. In the C57Bl/6 mice the inguinal node decreased in volume significantly between day 7 and day 28 (p = 0.0071) (7A). In nude mice the axillary, inguinal and popliteal nodes increased in volume over time; the axillary (p = 0.010) and inguinal nodes (p = 0.016) were significantly larger at day 28 compared to day 7 and the popliteal node was significantly larger at day 14 compared to day 7 (p = 0.0046) (7B). In SCID mice, the volumes of the brachial and inguinal nodes decreased significantly over time. The brachial (p = 0.013) and inguinal nodes (p = 0.023) were significantly smaller at day 14 compared to day 7. There were no significant differences in the volumes of the axillary and popliteal lymph nodes over time (7C).

## Discussion

This is the first study to use MRI to assess the appearance of the lymph nodes and spleen in various immuno-deficient and wild-type mouse strains. Given the substantial differences in the cellular composition of the immune systems in these mice, and reports from a small number of pathological studies which noted differences in lymph node morphology between different mouse strains, we hypothesized that the MRI appearance of the lymphoid tissues would also be disparate in the immuno-deficient and wild-type mouse strains we examined. MRI revealed some considerable differences in the appearance of lymph nodes in the different mice. Most notably, some lymph nodes in nude and SCID mice appeared with a region of signal hyperintensity in bSSFP images. By comparing the bSSFP images with T1- and T2-weighted SE images we were able to determine that the high signal intensity in the bSSFP images is most likely due to the presence of fluid within the node. Fluids have long T1 and T2 relaxation times. In T1-weighted images tissues with long T1 relaxation times appear dark, while in T2-weighted images tissues with long T2 relaxation times appear bright. Contrast in bSSFP images is related to T2/T1 and tissues with similar T1 and T2 values, like fluids (and fat), will have T2/T1 close to 1, which results in high signal intensity. It is also worth noting that when nude mice were followed over time with MRI, those nodes that appeared with a region of signal hyperintensity did not change in appearance during the imaging experiment.

There are relatively few papers that describe the anatomical or cellular features of normal lymph nodes in immune-deficient mice. In a paper by Sainte-Marie and Peng [17], 8 types of lymph nodes, in 7 nude and 4 C57Bl/10 mice, were carefully assessed by histopathology. They showed that in nude mice the absence of the thymus greatly inhibits the development of the lymphocyte population in the cortex, creating a small cavity. In addition, in some mice a cyst of variable size was found in the axillary or brachial nodes, often near the hilus [17]. These findings are similar to our observations in nude mice by MRI, where fluid filled cavities were observed in the axillary, brachial and inguinal nodes.

A histological examination of the immune organs in SCID mice, by Ge et al., showed that the lymph nodes had no clear cortex and appeared to be totally devoid of lymphocytes [18]. These modifications of lymph node anatomy could also be expected to result in features such as cavities or cysts. Our histology demonstrated that nodes which appeared with a region of high signal intensity in MR images, had a cavity within them, supporting the notion of a fluid-filled node.

Exposure of immuno-deficient mice to potentially pathogenic organisms must be restricted. Specific pathogen free (SPF) housing systems, often referred to as barrier facilities, are commonly used to house immuno-deficient mice and employ sterilization of feed, bedding, water, and cages along with the use of filter-top or individually ventilated caging systems and strict adherence to aseptic techniques for animal handling. Lymph nodes may enlarge when immune cells react to pathogen, such as virus or bacteria, due to proliferation of lymphocytes. Swollen glands, common to many illnesses, are an example of nodes enlarging in response to a pathogen. The immuno-deficient mice used in our longitudinal imaging studies leave the barrier facility for the first scanning session (day 7 after arrival) and thereafter were housed within an external barrier in ventilated cages. They were transported to/from the external barrier and the MRI facility for the next two scans (days 14 and 28). We hypothesized that the transfer between barriers and time spent outside of the barrier in the MRI facility, would result in changes in the size of the lymph nodes. To test this we imaged wild-type, nude and SCID mice at three time-points and measured the lymph node volumes. Changes in the lymph node volumes were measured for all mice. The largest changes were observed in the nude mice, however, the node volume in nude mice was found to be the most variable for all scans. In SCID mice the node volume was actually observed to decrease over time. However, there was no trend for increasing node size with number of times imaged (or number of transfers out of the barrier).

Even though the lymph nodes in these immuno-deficient mice are underdeveloped, or rudimentary, many studies show that the lymph nodes are a frequent site of cancer metastases [11], [19], [20]. In fact several studies suggest that lymph node metastases are more common in the mice with the more severe immunodeficiencies [12], [13]. Dewan et al. have reported that the rate of metastasis of human breast cancer cells (MDA-MB-231) is much higher in NOG mice, compared to NOD/SCID mice inoculated in the same way [12]. This included metastasis to the regional lymph nodes. It is interesting that, even though lymph nodes in NOG mice are not obvious at dissection, and not visible in MR images, the rudimentary node tissue still provides a suitable microenvironment for metastatic growth.

When lymph nodes are abnormal they increase in size [21]. Enlarged lymph nodes are readily visible in MRI. In fact, traditionally, MRI of the lymphatic system has been focused on conventional anatomical imaging whereby enlargement of lymph nodes is considered the primary diagnostic criterion for disease. Secondary architectural and pathological changes are also often apparent on MRI. It is therefore important to recognize that, in the different mouse strains we imaged, the size and appearance of the lymph nodes is quite variable in healthy animals and the lymph node volumes change over time in both wildtype and immuno-deficient mouse strains. A change in the lymph node size, as measured by MRI, in these mice should not be considered evidence of disease without additional validation.

In diseased lymph nodes the tissue is sometimes homogenized so that the cortical and medullary areas are no longer differentiated [22]. Necrosis may lead to accumulation of fluid (pus) within nodes, and can cause a fluid filed cavity. It is therefore very important to recognize that some diseased nodes can appear hyperintense in bSSFP and T2-weighted images (or with low signal in a T1-weighted image) and that this has the potential to be confused with normal lymph nodes in non-tumor bearing immuno-deficient mice.

The MRI appearance of the spleen in the different mouse strains was also notable. The spleens of wild-type and nude mice were quite similar in size and MRI appearance; a distinct architectural pattern is observed in MR images of the spleen in wild-type and nude mice. In SCID and NOG mice, the images show a smaller spleen (3–5x smaller in volume) devoid of pattern and in the case of the NOG mouse acellular. These differences in the MRI appearance likely reflect the impaired development of the spleen in these mice.

The normal spleen is composed of what is known as (non-lymphoid) red pulp and (lymphoid) white pulp. Red pulp consists of connective tissue and many splenic sinuses that are engorged with blood, giving it a red appearance. It functions to filter the blood and is a storage site for red blood cells [23]. It is also a reserve site for monocytes, which upon injury or disease leave the spleen and migrate to tissues for repair [23]. The high blood content causes it to appear with very low signal intensity in bSSFP images. White pulp consists of lymph nodules (germinal centers), composed of follicles, and periarteriolar lymphoid sheaths. White pulp is rich in lymphocytes [23].

SCID mice contain a defect preventing the functional development of T- and B- lymphocytes [24], [25]. Ge et al. have shown by histology that SCID mice have relatively empty splenic follicles [18]. It is because of these deficits the spleen in SCID mice is rudimentary in appearance and function. By MRI it appears to have no tissue contrast within it, suggesting minimal structural features. The NOG mouse is a SCID mouse strain that has multi-functional defects in NK activity, macrophage function, complement activity and dendritic cell function, in addition to lacking T- and B-cells. In all NOG mice examined by MRI the spleen appeared black reflecting an absence of signal likely due to an absence of cellularity.

In summary, this paper investigates an important technical aspect of mouse body imaging, namely the differential appearance of lymph nodes and spleens in 4 commonly used strains of experimental mice (C57Bl/6, nu/nu, CB-17 SCID, and NOG). These strains of mice are widely used for cancer research, and imaging is often used to identify metastasis to lymph nodes and distant organs. The use of these mice is not standardized; different laboratories use certain mice for a variety of reasons, and differences in the appearance of the lymph nodes across the strains can be a source of confusion in data interpretation. We have shown that there are particular features within the nodes of some mice that can mimic the appearance of pathology. We have found that changes in the size of nodes, in healthy mice, that occur with repeated imaging fall within the typical range of node sizes which show variability. By presenting knowledge of the normal MRI appearance of the lymphoid organs in healthy, immuno-deficient and immuno-competent mice we provide information that will help to avoid data misinterpretation and to advance the field.

## References

[1] Hudson W, Li Q, Le C, Kersey J (1998). Xenotransplantation of human lymphoid malignancies is optimized in mice with multiple immunologic defects.. Leukemia.

[2] Clarke R (1996). Human breast cancer cell line xenografts as models of breast cancer—the immunobiologies of recipient mice and the characteristics of several tumorigenic cell lines.. Breast Cancer Res Tr.

[3] Croy B, Linder K, Yager J (2001). Primer for Non-immunologists on Immune-Deficient Mice and Their Applications in Research.. Comparative Med.

[4] Shultz LD, Schweitzer PA, Christianson SW, Cott B, Schweitzer IB (1995). Multiple Defects in Innate and Adaptive Immunologic Function in NOD/LtSz-scid Mice.. J Immunol.

[5] Greiner DL, Shultz LD, Yates J, Appel MC, Perdrizet G (1995). Improved Engraftment of Human Spleen Cells in NOD/LtSz-scid/scid Mice as Compared with C.B-17-scid/scid Mice.. Am J Pathol.

[6] Ito M, Hiramatsu H, Kobayashi K, Suzue K, Kawahata M (2002). NOD/SCID/gamma null c mouse: an excellent recipient mouse model for engraftment of human cells.. Blood.

[7] Ito M, Kobayashi K, Nakahata T (2008). NOD/Shi-scid IL2r(gamma)null (NOG) Mice More Appropriate for Humanized Mouse Models.. Cur Top Microbiol Immunol.

[8] Brunner N, Boysen B, Romer J, Spang-thomsen M (1993). The nude mouse as an in vivo model for human breast cancer invastion and metastasis.. Breast Cancer Res Tr.

[9] Troiani T, Schettino C, Martinelli E, Morgillo F, Tortora G (2008). The use of xenograft models for the selection of cancer treatments with the EGFR as an example.. Crit Rev Oncol Hemat.

[10] Mukhopadhyay R, Theriault RL, Price JE (1999). Increased levels of α6 integrins are associated with the metastatic phenotype of human breast cancer cells.. Clin Exp Metastas.

[11] Price JE, Polyzos A, Zhang RD, Daniels LM (1990). Tumorigenicity and Metastasis of Human Breast Carcinoma Cell Lines in Nude Mice.. Cancer Res.

[12] Dewan MZ, Terunuma H, Ahmed S, Ohba K, Takada M (2005). Natural killer cells in breast cancer cell growth and metastasis in SCID mice.. Biomed Pharmacother.

[13] Mikhailov AD, Malakhov AA, Revazova ES, Valyakina TI, Yudicheva TV (1995). Metastasizing of Human Melanoma on Immunodeficient Mice. Comparison of Cell Lines with Different Metastasizing Activity.. B Exp Biol Med.

[14] Xie X, Brünner N, Jensenl G, Albrectsen J, Gotthardsen B (2000). Comparative studies between nude and scid mice on the growth and metastatic behavior of xenografted human tumors.. Clin Exp Metastas.

[15] Taghian A, Budach W, Zietman A, Freeman J, Gioioso D (1993). Quantitative Comparison between the Transplantability of Human and Murine Tumors into the Subcutaneous Tissue of NCr/Sed-nu/nu Nude and Severe Combined Immunodeficient Mice.. Cancer Res.

[16] Rosset A, Spadola L, Ratib O (2004). OsiriX: an open-source software for navigating in multidimensional DICOM images.. J Digit Imaging.

[17] Sainte-Marie G, Peng F (1983). Structural and cell population changes in the lymph nodes of the athymic nude mouse.. Lab Invest.

[18] Ge W, Bo C, Yan L, Xiaojuan Z, Kexun X (1991). Observation on husbandry and reproduction of mice with severe combined immunodeficiency and histological examination of their immune organs.. Chin J Cancer Res.

[19] Matsui J, Funahashi Y, Uenaka T, Watanabe T, Tsuruoka A (2008). Multi-Kinase Inhibitor E7080 Suppresses Lymph Node and Lung Metastases of Human Mammary Breast Tumor MDA-MB-231 via Inhibition of Vascular Endothelial Growth Factor-Receptor (VEGF-R) 2 and VEGF-R3 Kinase.. Clin Cancer Res.

[20] Dadiani M, Kalchenko V, Yosepovich A, Margalit R, Hassid Y (2006). Real-time imaging of lymphogenic metastasis in orthotopic human breast cancer.. Cancer Res.

[21] Barrett T, Choyke PL (2006). Kobayashi H. Imaging of the lymphatic system: new horizons.. Contrast Med Mol Imaging.

[22] Hutter R (1984). Pathological parameters useful in predicting prognosis for patients with breast cancer.. Monogr Pathol.

[23] Cesta MF (2006). Normal structure, function, and histology of the spleen.. Toxicol Pathol.

[24] Sculer W, Bosma MJ (1989). Nature of the scid defect: a defective VDJ recombinase system.. Cur Top Microbiol Immunol.

[25] Bosma GC, Custer RP, Bosma MJ (1983). A severe combined immunodeficiency mutation in the mouse.. Nature.

